# C-peptide and Central Nervous System Complications in Diabetes

**DOI:** 10.1080/15438600490424550

**Published:** 2004

**Authors:** Zhen-guo Li, Anders A. F. Sima

**Affiliations:** 1 Department of Pathology Wayne State University School of Medicine Room 9275 H.G. Scott Hall, 540 East Canfield Avenue Detroit Michigan 48201 USA; 2 Department of Neurology Wayne State University School of Medicine Detroit Michigan USA; 3 Morris J. Hood Jr. Comprehensive Diabetes Center Wayne State University School of Medicine Detroit Michigan USA

## Abstract

Substantial evidence collected from clinical data and experimental studies has indicated that CNS is not spared from diabetes complications. Impairments in CNS function are well documented in both type 1 and type 2 diabetic patients as well as in various animal models of diabetes, in terms of alterations in cognition, neuropsychology, neurobehavior, electrophysiology, structure, neurochemistry and apoptotic activities. These data suggest that *primary diabetic encephalopathy* exists as a definable diabetic complication. The mechanisms underlying this CNS complication are not clear. Experimental studies have suggested that neuronal apoptosis may play an important role in neuronal loss and impaired cognitive function. In diabetes multiple factors are responsible for neuronal apoptosis, such as a perturbed IGF system, hyperglycemia and the aging process itself. Recent data suggest that insulin/C-peptide deficiency may exert an eminent role. Administration of C-peptide partially corrects the perturbed IGF system in the brain and prevents neuronal apoptosis in hippocampus of type 1 diabetes. In neuroblastoma SH-SY5Y cells C-peptide provides a dose-dependent stimulation on cell proliferation and an anti-apoptotic effect as well. These studies provide a basis for administration of C-peptide as a potentially effective therapy for type 1 diabetes.


*Experimental Diab. Res.*, 5:79–90, 2004
Copyright © Taylor and Francis Inc.
ISSN: 1543-8600 print / 1543-8619 online
DOI: 10.1080/15438600490424550

### C-peptide and Central Nervous System Complications in Diabetes

**Zhen-guo Li[1,3] and Anders A. F. Sima[1,2,3]**

*Departments of [1]Pathology, [2]Neurology, and [3]Morris J. Hood Jr. Comprehensive Diabetes Center, Wayne State University School of Medicine, Detroit, Michigan, USA*

**Substantial evidence collected from clinical data and experimental studies has indicated that CNS is not spared from diabetes complications. Impairments in CNS function are well documented in both type 1 and type 2 diabetic patients as well as in various animal models of diabetes, in terms of alterations in cognition, neuropsychology, neurobehavior, electrophysiology, structure, neurochemistry and apoptotic activities. These data suggest that *primary diabetic encephalopathy* exists as a definable diabetic complication. The mechanisms underlying this CNS complication are not clear. Experimental studies have suggested that neuronal apoptosis may play an important role in neuronal loss and impaired cognitive function. In diabetes multiple factors are responsible for neuronal apoptosis, such as a perturbed IGF system, hyperglycemia and the aging process itself. Recent data suggest that insulin/C-peptide deficiency may exert an eminent role. Administration of C-peptide partially corrects the perturbed IGF system in the brain and prevents neuronal apoptosis in hippocampus of type 1 diabetes. In neuroblastoma SH-SY5Y cells C-peptide provides a dose-dependent stimulation on cell proliferation and an anti-apoptotic effect as well. These studies provide a basis for administration of C-peptide as a potentially effective therapy for type 1 diabetes.**

**Keywords** C-peptide Deficiency; Diabetes; Hyperglycemia; Insulin Deficiency; Neuronal Apoptosis; Primary Encephalopathy

Received 2 September 2003; accepted 28 November 2003.
Address correspondence to Anders A. F. Sima, MD, PhD, Department of Pathology, Wayne State University, Room 9275, H.G. Scott Hall, 540 East Canfield Avenue, Detroit, MI 48201, USA. E-mail: asima@med.wayne.edu

### INTRODUCTION

Diabetes is a common metabolic disorder affecting many systems, including muscle, retina, kidney, blood vessels (small and large vessels), and the nervous system. With respect to the nervous system, the peripheral nervous system (PNS) has traditionally been considered as the sole nervous system complication in diabetes, whereas the central nervous system (CNS) was believed to be spared from diabetes. However, recent studies have suggested that diabetes causes primary as well as secondary impairments in CNS function. In this review, we will describe the CNS complications occurring in diabetic patients and experimental models of diabetes. Several possible mechanisms will be discussed, although we will focus on neuronal apoptosis and CNS dysfunction.

### CNS COMPLICATIONS IN DIABETES

### Clinical Studies

Diabetes-related cognitive dysfunction has been recognized since the early 1920s in the medical literature (Miles and Root, 1922). Subsequently, many studies of both type 1 and type 2 diabetic subjects have found that diabetes mellitus is associated with a series of neurobehavioral or neuropsychological changes. "Diabetic encephalopathy" could, therefore, be included as one of the complications associated with diabetes.

#### *CNS Complications in Type 1 Diabetic Patients*

Impairments in learning, memory, problem solving, and mental and motor speed are more common in type 1 diabetic patients than in the general population (McCarthy et al., 2002; Ryan, 1988; Ryan and Williams, 1993; Ryan et al., 1993). The cognitive impairments can be severe in rare cases (Deary et al., 1993; Gold et al., 1994), but is in most cases modest. The variation in severity and pattern of impairment may be due to the subtle nature of the defects and variation in the diabetic populations selected for study, the patient number in each study, the psychological tests employed, and variations in glycemic controls among individual studies. A lower Wechsler Intelligence Scale for Children—Revised (WISC-R) performance IQ was found in type 1 diabetic children with disease onset at less than 7 years of age and disease duration greater than or equal to 5 years (Holmes and Richman, 1985). Diabetic patients suffered from significantly more introversive symptoms than their healthy counterparts, especially with respect to somatic symptoms, sleeping disturbances, compulsions, and depressive moods (Blanz et al., 1993; Kovacs et al., 1997; Lustman et al., 1988; Popkin et al., 1988). These studies thus indicate impaired CNS function in type 1 diabetes.

The neuropsychological changes are often accompanied with objective electrophysiological, structural, and neurochemical abnormalities. In electrophysiological studies, measurements of latencies of evoked potentials have been widely used to examine the functional integrities of the CNS in diabetic patients (Di Mario et al., 1995). Delayed conduction velocity and data-processing time in the CNS were demonstrated in type 1 diabetic patients, including brainstem auditory-evoked potential, somatosensory-evoked potentials, visual-evoked potentials, and motor-evoked potentials (Comi, 1997; Cracco et al., 1984; Harkins et al., 1985; Pietravalle et al., 1993; Uberall et al., 1996; Verrotti et al., 2000). The latency of the P100 wave of the visual-evoked potential, which is believed to be generated in the visual cortex, is increased in diabetic patients (Parisi and Uccioli, 2001; Ziegler et al., 1994). The event-related (P300) potential is an objective measure of cognitive function (Madl et al., 1994; Picton, 1992). Several studies have shown that P300 latency, a neurophysiological correlate of information processing, such as stimulus evaluation, alertness, and memory updating (Kramer et al., 1998), is increased in diabetic patients (Kramer et al., 1998; Pozzessere et al., 1991; Uberall et al., 1996), indicating an impairment of higher brain functions.

In terms of structural changes, several abnormalities have been described in type 1 diabetic patients, such as diffuse and local degenerative changes of cerebral cortex (Reske-Nielsen and Lundbaek, 1964; Reske-Nielsen et al., 1965), neuronal loss, demyelination and gliosis, and infarction secondary to microangiopathy (DeJong, 1977). Magnetic resonance imaging and computed tomography have shown that widened sulci and/or enlarged lateral ventricles (Araki et al., 1994; Lunetta et al., 1994; Perros et al., 1997) and increased occurrence of white matter hyperintensities (Araki et al., 1994; Perros et al., 1997) are more pronounced in diabetic patients than in age-matched control subjects. These studies indicate structural changes in CNS of diabetes.

Because hypoglycemia itself can cause brain damages (de Courten-Myers et al., 2000; Vannucci and Vannucci, 2001; Yager, 2002), it remains controversial as to whether the above CNS abnormalities are caused by diabetes per se (primary) or are the result of hypoglycemic episodes (secondary), which occur frequently in type 1 diabetic patients receiving intensive insulin treatment. Recent studies have shown that a duration-dependent decline in cognitive function occurs in type 1 diabetic patients without hypoglycemic episodes (Kramer et al., 1998), and that impaired intellectual and cognitive development occur in type 1 diabetic children, who have not experienced hypoglycemic episodes (Schoenle et al., 2002). These latter impairments correlate with diagnosis at young age, male sex, and metabolic status at time of diagnosis. Therefore, CNS complications seem to be impacted by diabetes itself without implementing hypoglycemic episodes as a causative mechanism and hence being primary.

In summary, evidence obtained from pathologic, physiologic, psychologic, and behavioral studies point toward a "*primary diabetic encephalopathy*" in type 1 diabetes.

### *CNS Complications in Type 2 Diabetic Patients*

Neuropsychologic studies in type 2 diabetes patients have shown more consistent results compared to type 1 diabetes patients. Cognitive deficits (Gradman et al., 1993; Perlmuter et al., 1984; Ryan and Geckle, 2000a; Strachan et al., 1997; Tun et al., 1990; Worrall et al., 1993) and poor performance in abstract reasoning and complex psychomotor functioning (Reaven et al., 1990) occur in type 2 diabetes. Complex cognitive functions, as demonstrated by complex cognitive tasks requiring storage and retrieval of new information, are affected, whereas performances of less demanding tasks, such as immediate memory and simple reaction time, are not significantly altered (Tun et al., 1990). Electrophysiological studies have demonstrated delayed CNS conduction velocity (Donald et al., 1984; Varsik et al., 2001; Verrotti et al., 2000) and increased latency of P100 (Ziegler et al., 1994). Several studies demonstrate that P300 latency is increased in type 2 diabetic patients (Dey et al., 1997; Kurita et al., 1995; Mochizuki et al., 1998; Mooradian et al., 1988), indicating an impairment of higher brain function. Recently, increased blood-brain barrier (BBB) permeability in type 2 diabetes was demonstrated by magnetic resonance imaging when intravenous administration of gadolinium diethylenetriamine pentaacetic acid (Gd-DTPA) was used to identify BBB permeability, suggesting that loss of BBB integrity may play a role in CNS dysfunction in diabetes (Starr et al., 2003). In the All Wales Research into Elderly (AWARE) Diabetes Study, elderly type 2 diabetic patients displayed significant excess of cognitive dysfunction associated with a poorer ability in diabetes self-care (Sinclair et al., 2000). However, no significant impairments in learning, memory and problem-solving skills were found in middle-aged type 2 diabetic patients compared to control subjects (Ryan and Geckle, 2000b). In fact, few studies have been conducted regarding cognitive deficits in middle-aged type 2 diabetic patients, among which the largest patient number was 80 type 2 diabetic patients versus 81 nondiabetic controls (Lowe et al., 1994). Therefore, more studies with large number of patients are needed in determining cognitive function in middle-aged type 2 diabetes. The current thought is that learning and memory dysfunction are more prominent in elderly type 2 diabetic subjects (Ryan and Geckle, 2000a). As to whether this is due to a potentiation of the normal aging process by superimposed diabetes or a function of diabetes duration alone is not settled.

The etiology of cognitive dysfunction in type 2 diabetes is not fully understood. It is likely that cognitive impairments are caused by an interaction between metabolic abnormalities intrinsic to diabetes and diabetes-specific complications. After all, the higher frequency of cerebral stroke in type 2 diabetes may be associated with cognitive deficits. Several studies have suggested that the cerebrovascular mortality rate is higher in patients with type 2 diabetes (Barrett-Connor and Khaw, 1988; Lehto et al., 1996). The risk for developing stroke is increased 2- to 5-fold compared to nondiabetic control subjects (Manson et al., 1991; Stamler et al., 1993). Recently, 5102 patients with type 2 diabetes in the UK Prospective Diabetes Study (UKPDS) were analyzed using a newly developed mathematical model. The results confirmed the higher risk of stroke in type 2 diabetes (Kothari et al., 2002). Although cerebrovascular disease is barely mentioned in patients with type 1 diabetes, recent analyses of mortality from 23,000 patients with type 1 diabetes has shown for the first time that cerebrovascular mortality is raised at all ages in this patient group (Laing et al., 2003). Therefore, type 1 diabetes is at least as great a risk factor for cerebrovascular mortality as is type 2 diabetes. Based on these reports, the continuing study of cerebral stroke in diabetes will provide useful insight into possible underlying mechanisms.

### Experimental Studies

### *Structural Changes*

A variety of structural changes has been described in the CNS of streptozotocin (STZ)-induced diabetes in rats and Chinese hamsters (Bestetti and Rossi, 1980, 1982; Garris et al., 1982; Jakobsen et al., 1987; Luse, 1970; Mukai et al., 1980). Structural alterations of the ventromedial hypothalamus, including accumulation of glycogen, degeneration of neurons, and atrophy of tanycytes, have been described. Ultrastructural abnormalities, such as dilated and fragmented endoplasmic reticulum, degranulated ergastoplasm, increased number of microtubuli, myelin figures, irregularities in the form of nuclei, and appearance of chromatin, were described. A significant loss of neocortical neurons occur in STZ-diabetic rats compared to nondiabetic control rats (Jakobsen et al., 1987). In addition, significant structural alterations in the BBB were shown in experimental diabetic animals (Mooradian, 1997), consistent with the magnetic resonance imaging findings in diabetic patients (Starr et al., 2003).

### *Neurochemical Changes*

The concentration of neurotransmitters appear to be altered in diabetic brains. A decrease in the norepinephrine (NE) and serotonin (5-OT) content was shown in the neocortex and caudal segment of the brain stem in alloxan-induced diabetic rats (Kulikov et al., 1986) and in brains of STZ-induced diabetic rats (Trulson et al., 1986). In contrast, an increase in NE concentrations in the paraventricular nucleus, lateral hypothalamus, ventromedial hypothalamus, and suprachiasmatic nucleus and increased concentrations of NE, dopamine, and serotonin in the arcuate nucleus have been described in STZ-diabetic rats (Barber et al., 2003), indicating that the monoaminergic system is affected in experimental diabetic animals. In this study, insulin treatment reversed these changes, suggesting that insulin deficiency and/or hyperglycemia may play a role in the alteration of monoamine neurotransmission.

### *Electrophysiological Changes*

The latencies of auditory and visual potentials were found to be prolonged in STZ-diabetic rats (Biessels et al., 1999; Rubini et al., 1992) and BB/W diabetic rats (Chakrabarti et al., 1991; Sima et al., 1992), indicating impaired CNS conduction velocities in diabetes. In hippocampal slices from STZ-diabetic rats, long-term potentiation (LTP) induced by 100-Hz stimulation was impaired, whereas long-term depression (LTD) was enhanced compared with control rats (Biessels et al., 1998; Gispen and Biessels, 2000; Kamal et al., 2000), indicating that altered hippocampal synaptic plasticity occurs in type 1 STZ-diabetic rats. Because changes in hippocampal synaptic plasticity in STZ-diabetic rats occur in association with impairments of spatial learning function (Gispen and Biessels, 2000; Kamal et al., 2000), these studies provide a mechanism for cognitive dysfunction in diabetic animals.

### *Cognitive Functional Changes*

Impaired spatial learning and memory have been demonstrated using the Morris water maze system in various diabetic animals including BB/W rats, STZ-diabetic rats, STZ-diabetic mice, and Otsuka Long Evans Tokushima Fatty (OLETF) rats (Biessels et al., 1998; Flood et al., 1990; Kamal et al., 2000; Li et al., 2002a, b; Luesse et al., 2001). Multiple cognitive components are involved in this task, such as problem solving, enhanced selective attention, formation of internal representation of the external environments, and storage and retrieval of relevant information (Bannerman et al., 1995). The hippocampal LTP and LTD are two forms of neuronal synaptic plasticity. In young adult STZ-diabetic rats, hippocampal LTP expression is impaired, whereas LTD expression is enhanced (Biessels et al., 1996; Kamal et al., 1999). The alterations in hippocampal synaptic plasticity is associated with defects in spatial learning and memory as detected by the Morris water maze (Biessels et al., 1996, 1998). The impaired performance in the Morris water maze started 10 weeks after the injection of STZ (Biessels et al., 1998; Kamal et al., 2000) and the degree of LTP impairment correlated with the duration of diabetes (Kamal et al., 1999). In diabetic BB/W rats, we failed to show impaired spatial learning and memory in acutely diabetic rats (2-month), whereas significant deficits in the Morris water maze were demonstrated in 8-month diabetic BB/W rats (Li et al., 2002b). These results suggest that progressive learning and memory deficiencies in type 1 diabetic rats occur in a duration-related fashion.

*Neuronal Loss/Apoptosis in Type 1 Diabetes*

A significant loss of neocortical neurons occurs in STZ-diabetic rats (Jakobsen et al., 1987). In the diabetic Chinese hamster, neuronal death occurs in the arcuate and ventromedial nuclei (Garris, 1984). The nature of cell death was not defined in these early studies, nor was it determined as to whether neuronal loss was associated with cognitive deficits. In the spontaneously diabetic BB/Wor rat, neuronal counts of hippocampal pyramidal cells were performed in the hippocampal $CA_1$–$CA_4$ regions. No significant differences were demonstrated at 2 months of diabetes. In 8-month diabetic animals, there were a 37% and 24% loss of pyramidal cells in $CA_1$ and $CA_2$ regions, respectively, whereas other hippocampal regions showed non-significant decreases in neuronal densities (Li et al., 2002b). To exclude the effect of body weight on neuronal density, we compared 8-month diabetic rats with weight-matched control rats, which did not hippocampal neuronal loss. These data suggest that decreased neuronal density is not weight related but diabetes duration related. Comparisons with hyperglycemia- and duration-matched spontaneously type 2 diabetic BBDRZ/Wor rats revealed a much milder but still significant neuronal loss of the hippocampal $CA_1$ region (Li and Sima, unpublished data).

The above studies indicate that neuronal loss occurs in type 1 diabetic animals. As to the nature of this neuronal loss, we have proposed that apoptosis might play an important role. Several in vitro and in vivo studies appear to support this notion (Li et al., 2002b, 2003). No apoptotic activity of hippocampal pyramidal neurons was shown in 2-month diabetic BB/Wor rats, nor was any cognitive deficits detected by the Morris water maze procedure, nor was any neuronal loss detectable. On the other hand, in 8-month diabetic rats, TUNEL-positive neurons, positive DNA laddering, and increased Bax expression and caspase-3 activity were evident and associated with decreased neuronal density and impaired Morris water maze performances (Li et al., 2002b). Hence, duration-related apoptosis is likely to account for the neuronal loss and the concomitant emergence of cognitive impairments in the type 1 diabetic BB/Wor rat.

*Ischemic Injury in Diabetic Animals*

It is well known that diabetes aggravates brain damage in experimental and clinical stroke subjects. Diabetes accelerates maturation of neuronal damage, increases infarct volume, and induces postischemic seizures (Muranyi et al., 2003). A brief period of 30 minutes' focal ischemia in normoglycemic rats leads to brain damage in a delayed fashion: infarction develops after 3 days and full maturation is reached by 2 weeks after recirculation (Du et al., 1996). If preischemic hyperglycemia exists or in STZ-diabetic rats, the damage is more severe than that in nondiabetic animals (Muranyi et al., 2003) and the damage evolves faster: infarction develops after 2 hours and is matured after 4 to 6 hours after the ischemic insult (Gisselsson et al., 1999; Li et al., 1998). The mechanisms underlying diabetes-related aggravation of ischemic brain damage are unclear. Apoptosis, or programmed cell death (PCD), is believed to play an important role in the pathogenesis of various human disorders including diabetes (Barr and Tomei, 1994; Fadeel et al., 1999; Orrenius, 1995; Thompson, 1995) and a vast body of studies has shown that apoptosis is associated with cerebral ischemia and ischemia-reperfusion injury. It has been demonstrated that a large number of neurons bordering the maturing infarct exhibit prominent TUNEL staining, and DNA prepared from the penumbral area of ischemic cortex show internucleosomal fragmentation (Du et al., 1996). Increased levels of either expression or activities of caspase-3 (Chen et al., 1998; Schulz et al., 1999) and induction of caspase-activated deoxyribonuclease activity (Luo et al., 2002) are detected in ischemic neurons. DNA fragmentation in ischemic brains has been demonstrated by either gel electrophoresis or TUNEL staining (Charriaut-Marlangue et al., 1998; Endres et al., 1998; Li et al., 1995). A strong correlation has been found among caspase-3 activity, TUNEL staining, and the appearance of apoptotic neurons (Cao et al., 2001; Charriaut-Marlangue et al., 1998; Chen et al., 1998; Namura et al., 1998). Furthermore, cytosolic cytochrome *c* release is markedly enhanced in both the ischemic focus and the penumbra in STZ-diabetic rats, together with increased activation of caspase-3 and accelerated cleavage of poly-ADP ribose polymerase (PARP) (Du et al., 1996), all of which suggest that apoptosis plays a role in the exaggerating effect of diabetes on cerebral ischemic lesions.

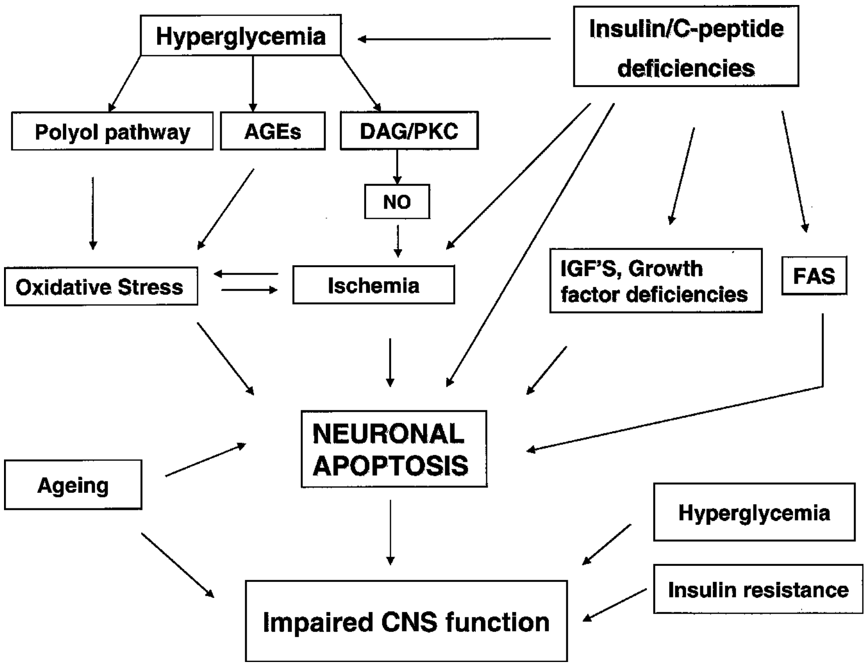

**FIGURE 1**
Possible pathways leading to CNS dysfunction in diabetes.

Recently, the effects of local ischemia on type 1 diabetic rats versus normal control rats were reported (Britton et al., 2003). A significant increase in the number of TUNEL-positive and caspase-3–positive cell was demonstrated in selected brain regions (hypothalamic preoptic area, piriform cortex, and parietal cortex) of STZ-diabetic rats subjected to middle cerebral artery occlusion for 24 to 48 hours, compared with nonoccluded diabetic rats (Britton et al., 2003). Consistent with these findings, preliminary data show that the ladder pattern of nucleosomal DNA fragmentation (ligation mediated–polymerase chain reaction [LM-PCR]) cannot be detected in unlesioned normal or STZ-diabetic animals, but is evident in infarcted cortex of diabetic rats (Li and Sima, unpublished data). Bax expression is increased in normal infarcted cortex and is further increased in infarcted diabetic cortex. These data suggest that diabetes confers an expansion of stroke via increased apoptotic activity.

In summary, data obtained from experimental type 1 diabetic animals show definite alterations in structure, neurotransmitters, electrophysiology, cognitive function, neuronal density, and apoptotic activity. These results clearly indicate that "*diabetic encephalopathy*" is an identifiable diabetic complication.

### POSSIBLE MECHANISMS

The mechanisms underlying neuronal apoptosis and CNS dysfunction are not clear. We believe that contributing factors are insulin deficiency with concomitant C-peptide deficiency, as well as hyperglycemia and possibly the aging process itself (Figure 1).

### Insulin/C-peptide Deficiency

Insulin plays an important role in regulation of brain metabolism. Recent studies show that insulin exerts additional modulatory roles on brain functions such as feeding (Gerozissis et al., 2001; Schwartz et al., 1992), learning, and memory (Gerozissis et al., 2001; Zhao and Alkon, 2001). In cerebral stroke, administration of insulin prevents neuronal death (Voll and Auer, 1991a; Voll and Auer, 1991b) and reduces neurological disability (LeMay et al., 1988). These neuroprotective effects of insulin may be associated with restoration of protein synthesis via increased dephosphorylation of eukaryotic iniation factor 2$\alpha$ (Sullivan et al., 1999). In STZ-diabetic rats, cognitive deficits is partially corrected by insulin (Biessels et al., 1998; Gradman et al., 1993). In vitro studies also show that insulin has an antiapoptotic effects (Bertrand et al., 1999; Lee-Kwon et al., 1998; Li et al., 2003). We have shown that the insulin receptor is downregulated in the brain of type 1 diabetic BB/Wor rats (Li et al., 2002b), suggesting that impaired insulin action caused not only by insulinopenia but also by impaired insulin receptor expression may play a role in CNS apoptosis in diabetes.

In addition to insulin deficiency, C-peptide deficiency has been considered as a pathogenic factor in type 1 diabetic

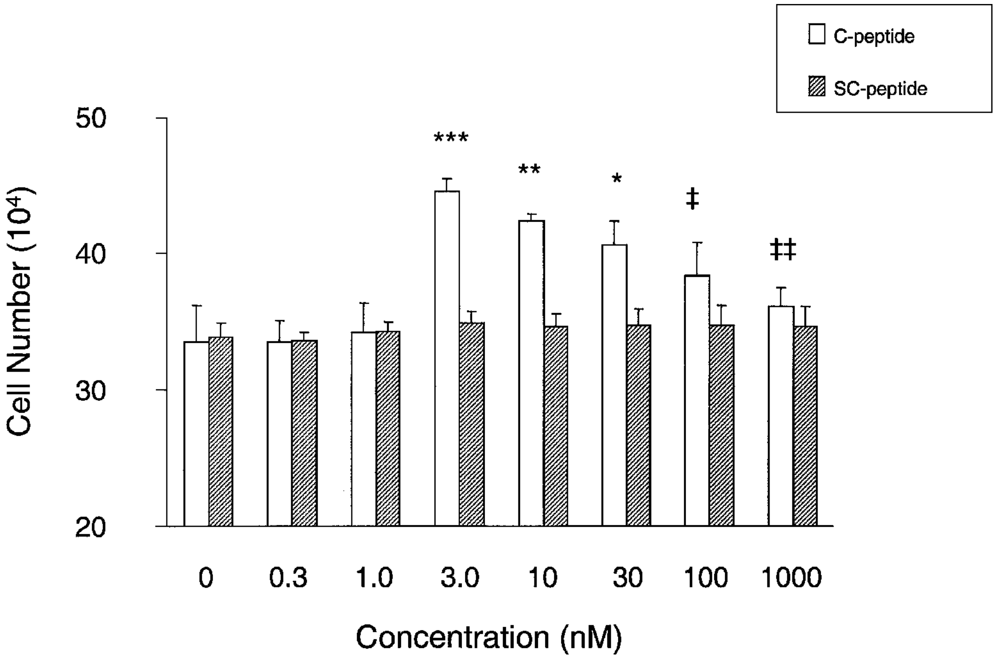

**FIGURE 2**

C-peptide promotes proliferation of SH-SY5Y cells. Maximal stimulatory effect of C-peptide was demonstrated at a concentration of 3 nM, whereas scrambled C-peptide did not show demonstrate any stimulatory effect on cell proliferation. Data are expressed as mean ± SD of 3 individual experiments. $^{*}P < .05$; $^{**}P < .01$; $^{***}P < .005$ versus control; $^{‡}P < .05$; $^{‡‡}P < .01$ versus 3 nM C-peptide.

complications. C-peptide is a 31–amino acid peptide that is cleaved from proinsulin during biosynthesis of insulin (Li et al., 2001; Steiner and Rubenstein, 1997). Recent studies have shown that C-peptide possesses physiological functions other than providing structural support for proinsulin cleavage (Wahren and Johansson, 1998; Wahren et al., 1994, 1996; Sima, 2003a, 2003b). In patients with type 1 diabetes, C-peptide improves renal function, reduces urinary albumin excretion and glomerular filtration, and decreases blood retinal barrier leakage (Fernqvist-Forbes et al., 2001; Forst et al., 1998; Johansson et al., 1992, 1996; Zierath et al., 1991, 1996). Chronic C-peptide replacement prevents functional and structural peripheral nerve changes in type 1 diabetic rat models (Huang et al., 2002; Ido et al., 1997; Li et al., 1999; Samnegard et al., 2001; Wu et al., 1996; Zhang et al., 2001; Sima et al., 2001; Pierson et al., 2003), suggesting that C-peptide deficiency is a participating factor in the causation of type 1 diabetic complications. We recently showed that administration of C-peptide partially corrects perturbed insulin-like growth factor (IGF) activity and prevents the neuronal apoptosis in hippocampus of type 1 diabetic BB/W rats (Li et al., 2002c), indicating a relationships between C-peptide deficiency, IGF perturbation, and neuronal apoptosis. In neuroblastoma SH-SY5Y cells, C-peptide provides a dose-dependent stimulatory effect on cell proliferation, whereas no effects were evident with the same concentration of scrambled C-peptide (Figure 2). In the same in vitro cell cultures, apoptosis is induced by high concentrations of glucose, mimicking hyperglycemia. Apoptosis of SH-SY5Y cells is inhibited by addition of insulin alone, whereas the combination of insulin and C-peptide show an enhanced antiapoptotic effect, as assessed by prevention of nuclear condensation and shrinkage, reduction in the number of apoptotic cells, and stimulation of Bcl2 expression and nuclear factor (NF)-$\kappa$B (Li et al., 2003). The inactivated form NF-$\kappa$B exists in the cytoplasm as a complex consisting of p50, p65, subunits of inhibitor-$\kappa$B (I$\kappa$B). On stimulation, I-$\kappa$B is phosphorylated and degraded, resulting in activation and translocation of NF-$\kappa$B to the nucleus, where it activates target gene transcription. It is known that NF-$\kappa$B and the genes regulated by this transcription factor, such as those coding for tumor necrosis factor (TNF) receptor–associated factor 1 (TRAF1), TRAF2, inhibitor-of-apoptosis (IAP) proteins c-IAP1 and c-IAP2, manganese superoxide dismutase (MnSOD), Bcl2, and BclxL, play important roles in the regulation of apoptosis (Aggarwal, 2000; Bours et al., 2000). In the SH-SY5Y cells grown under elevated glucose concentrations, C-peptide plus insulin gives rise to a significant increase in nuclear NF-$\kappa$B staining (Figure 3), suggesting that insulin/C-peptide play effective antiapoptotic roles via activation and translocation of NF-$\kappa$B. In summary, the data obtained from in vitro system are consistent with the results obtained from experimental diabetes

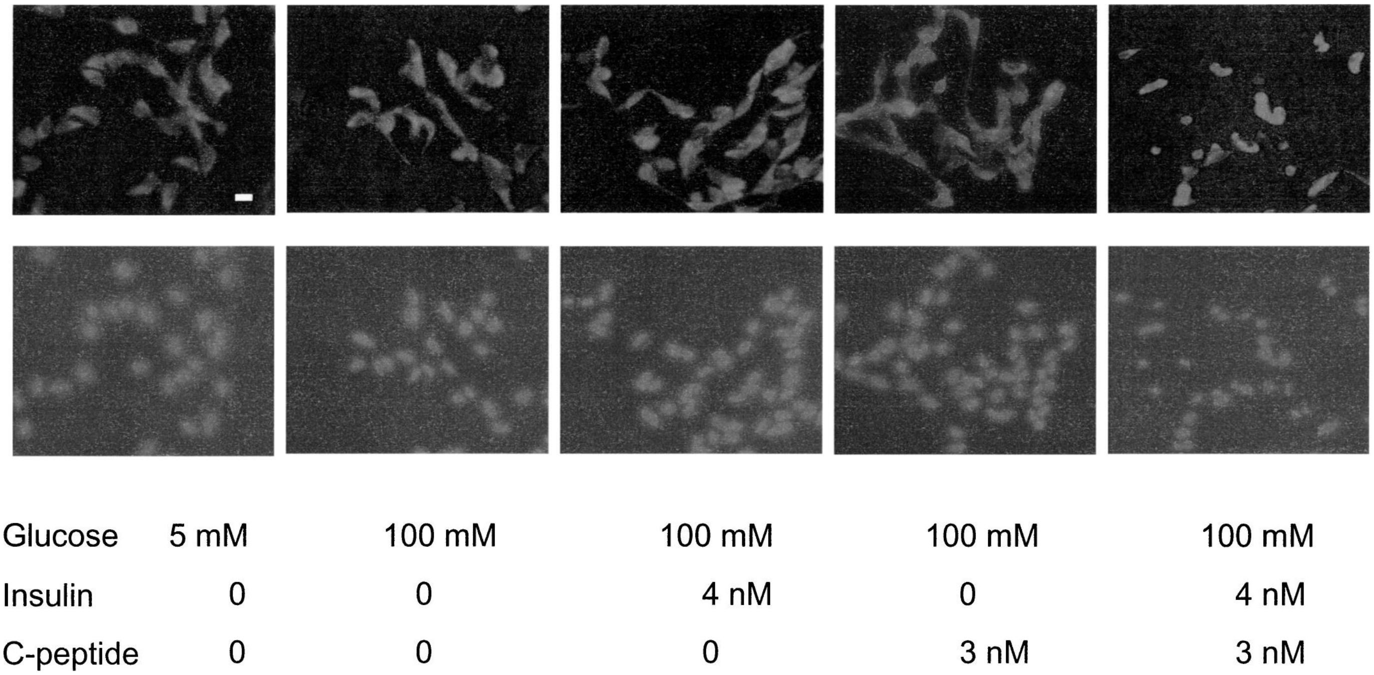

**FIGURE 3**
The effects of insulin and/or C-peptide on translocation of NF-κB in SH-SY5Y cells incubated in 100 mM glucose. Localization of NF-κB is shown by immunocytochemistry (*top panels*), whereas the content of nuclear NF-κB is matched to nuclear staining with DAPI (*bottom panels*), bar = 10 μm. Nuclear content of NF-κB was markedly increased when cells were exposed to the combination of 3 nM C-peptide and 4 nM insulin.

in animals and support the notion that insulin/C-peptide deficiency plays an important role in type 1 diabetes-induced neuronal apoptosis.

### IGF System Impairments

Perturbed IGF system has been shown in the CNS of STZ-diabetic rats. After 2 weeks of diabetes, IGF-II mRNA content is significantly decreased in brain and spinal cord. Insulin replacement partially restores IGF-II mRNA levels in cerebral, cortex, medulla, and spinal cord (Wuarin et al., 1996). We have systemically examined the IGF system (IGF-1, IGF-II, IGF-IR, and insulin receptor [IR]) in the BB/Wor model of type 1 diabetes. Using Western blotting, Northern hybridization, and in situ hybridization, we found significant reductions in the expression of IGF-I, IGF-II, IGF-IR, and IR already in 2-month diabetic BB/Wor rats, which persisted in 8-month diabetic rats, indicating that these abnormalities precede the functional cognitive impairments and the apoptotic neuronal loss in hippocampus (Li et al., 2002b).

### Hyperglycemia

Hyperglycemia causes increased glucose levels in the brain. It is well established that hyperglycemia causes oxidative stress via the polyol pathway, enhanced advanced glycation end products (AGEs), and increased lipid peroxidation by-products and imbalances in the generation of reactive oxygen species and their scavengers (Ceriello et al., 1993; Lipinski, 2001; Mercuri et al., 2000; Opara, 2002). Oxidative stress is also associated with cerebral ischemic injury (Kaminski et al., 2002; Love, 1999) and neuronal apoptosis (Gorman et al., 1996; Nicotera et al., 1997; Sastre et al., 2000). Extreme hyperglycemia also causes nonketotic hyperosmolar coma, characterized by volume depletion, altered consciousness, confusion, and, less frequently, neurological deficit in the absence of ketonemia or acidosis. Hyperosmolar coma is a severe complication of diabetes with a high overall mortality rate (Amundson et al., 1996; Braaten, 1987; Rolfe et al., 1995; Ting, 2001). It is therefore likely that hyperglycemia per se may induce apoptotic activities, preferably via oxidative stress, as indicated by hippocampal apoptosis with neuronal loss in the type 2 hyperinsulinemic BBDRZ/Wor rat (Li and Sima, unpublished data.

### Aging

Aging in developed countries is associated with an increasing prevalence of hypertension, atherosclerotic vascular diseases, reduced insulin sensitivity, and type 2 diabetes

(Barbagallo et al., 1997). In the Rotterdam Study, a community-based prospective cohort study, chronic disorders of the elderly were investigated. The relative risk for developing dementia was doubled in diabetic patients (Ott et al., 1999), suggesting that diabetes increases the risk of dementia, particularly in the elderly (Gardoni et al., 2002). It was recently demonstrated (Terry et al., 2001) that the expression of IGF-I, IGF-IR, and IR is reduced in susceptible hippocampal areas in Alzheimer's disease. We have shown that impaired IGF and insulin activities precede the development of hippocampal apoptosis in type 1 diabetic BB/W rats (Li et al., 2002b). The similarity in perturbations of the IGF system suggests that possible common etiological factors may be present in Alzheimer's disease and diabetes, two disorders in which apoptosis has been invoked as a mechanism. However, other studies have failed to find an association between diabetes and incident Alzheimer's disease (Hassing et al., 2002; MacKnight et al., 2002). More work is needed to elucidate the effect of aging on impaired CNS function in diabetes.

### CONCLUSION

Diabetes causes impairments in CNS functions. *Primary diabetic encephalopathy* is characterized by neurophysiological impairments, structural changes, and cognitive deficits. The mechanisms underlying this CNS dysfunction of diabetes are not fully elucidated. We propose that insulin/C-peptide deficiency in type 1 diabetes may be one important factor in the pathogenesis of CNS dysfunctions. It appears that apoptotic phenomena are contributing factors. However, much work is still needed to elucidate possible apoptotic pathways and likely alterations in contraregulatory mechanisms. The increased infarct size seen both in diabetic patients and animals following cerebral ischemia is likely to be caused by apoptosis. As to whether these apoptotic mechanisms are the same as those involved in *primary diabetic encephalopathy* remain to be examined.

### REFERENCES

Aggarwal, B. B. (2000) Apoptosis and nuclear factor-kappa B: A tale of association and dissociation. *Biochem. Pharmacol.,* **60,** 1033–1039.

Amundson, C. D., Olsen, C. J., and Wade, C. D. (1996) Partial central diabetes insipidus complicating nonketotic hyperglycemic hyperosmolar coma. *J. Am. Osteopathol. Assoc.,* **96,** 603–604.

Araki, Y., Nomura, M., Tanaka, H., Yamamoto, H., Yamamoto, T., Tsukaguchi, I., and Nakamura, H. (1994) MRI of the brain in diabetes mellitus. *Neuroradiology,* **36,** 101–103.

Bannerman, D. M., Good, M. A., Butcher, S. P., Ramsay, M., and Morris, R. G. (1995) Distinct components of spatial learning revealed by prior training and NMDA receptor blockade. *Nature,* **378,** 182–186.

Barbagallo, M., Resnick, L. M., Dominguez, L. J., and Licata, G. (1997) Diabetes mellitus, hypertension and ageing: The ionic hypothesis of ageing and cardiovascular-metabolic diseases. *Diabetes Metab.,* **23,** 281–294.

Barber, M., Kasturi, B. S., Austin, M. E., Patel, K. P., MohanKumar, S. M., and MohanKumar, P. S. (2003) Diabetes-induced neuroendocrine changes in rats: Role of brain monoamines, insulin and leptin. *Brain Res.,* **964,** 128–135.

Barr, P. J., and Tomei, L. D. (1994) Apoptosis and its role in human disease. *Biotechnology (NY),* **12,** 487–493.

Barrett-Connor, E., and Khaw, K. T. (1988) Diabetes mellitus: An independent risk factor for stroke? *Am. J. Epidemiol.,* **128,** 116–123.

Bertrand, F., Desbois-Mouthon, C., Cadoret, A., Prunier, C., Robin, H., Capeau, J., Atfi, A., and Cherqui, G. (1999) Insulin antiapoptotic signaling involves insulin activation of the nuclear factor kappaB-dependent survival genes encoding tumor necrosis factor receptor-associated factor 2 and manganese-superoxide dismutase. *J. Biol. Chem.,* **274,** 30596–30602.

Bestetti, G., and Rossi, G. L. (1980) Hypothalamic lesions in rats with long-term streptozotocin-induced diabetes mellitus. A semiquantitative light- and electron-microscopic study. *Acta Neuropathol. (Berl.),* **52,** 119–127.

Bestetti, G., and Rossi, G. L. (1982) Hypothalamic changes in diabetic Chinese hamsters. A semiquantitative, light and electron microscopic study. *Lab. Invest.,* **47,** 516–522.

Biessels, G. J., Cristino, N. A., Rutten, G. J., Hamers, F. P., Erkelens, D. W., and Gispen, W. H. (1999) Neurophysiological changes in the central and peripheral nervous system of streptozotocin-diabetic rats. Course of development and effects of insulin treatment. *Brain,* **122 (Pt 4),** 757–768.

Biessels, G. J., Kamal, A., Ramakers, G. M., Urban, I. J., Spruijt, B. M., Erkelens, D. W., and Gispen, W. H. (1996) Place learning and hippocampal synaptic plasticity in streptozotocin-induced diabetic rats. *Diabetes,* **45,** 1259–1266.

Biessels, G. J., Kamal, A., Urban, I. J., Spruijt, B. M., Erkelens, D. W., and Gispen, W. H. (1998) Water maze learning and hippocampal synaptic plasticity in streptozotocin-diabetic rats: Effects of insulin treatment. *Brain Res.,* **800,** 125–135.

Blanz, B. J., Rensch-Riemann, B. S., Fritz-Sigmund, D. I., and Schmidt, M. H. (1993) IDDM is a risk factor for adolescent psychiatric disorders. *Diabetes Care,* **16,** 1579–1587.

Bours, V., Bentires-Alj, M., Hellin, A. C., Viatour, P., Robe, P., Delhalle, S., Benoit, V., and Merville, M. P. (2000) Nuclear factor-kappa B, cancer, and apoptosis. *Biochem. Pharmacol.,* **60,** 1085–1089.

Braaten, J. T. (1987) Hyperosmolar nonketotic diabetic coma: diagnosis and management. *Geriatrics,* **42,** 83–88, 92.

Britton, M., Rafols, J., Alousi, S., and Dunbar, J. C. (2003) The effects of middle cerebral artery occlusion on central nervous system apoptotic events in normal and diabetic rats. *Int. J. Exp. Diabetes Res.,* **4,** 13–20.

Cao, G., Pei, W., Lan, J., Stetler, R. A., Luo, Y., Nagayama, T., Graham, S. H., Yin, X. M., Simon, R. P., and Chen, J. (2001) Caspase-activated DNase/DNA fragmentation factor 40 mediates apoptotic DNA fragmentation in transient cerebral ischemia and in neuronal cultures. *J. Neurosci.,* **21,** 4678–4690.

Ceriello, A., Quatraro, A., and Giugliano, D. (1993) Diabetes mellitus and hypertension: The possible role of hyperglycaemia through oxidative stress. *Diabetologia,* **36,** 265–266.

Chakrabarti, S., Zhang, W. X., and Sima, A. A. F. (1991) Optic neuropathy in the diabetic BB-rat. *Adv. Exp. Med. Biol.,* **291,** 257–264.

Charriaut-Marlangue, C., Remolleau, S., Aggoun-Zouaoui, D., and Ben-Ari, Y. (1998) Apoptosis and programmed cell death: A role in cerebral ischemia. *Biomed. Pharmacother.,* **52,** 264–269.

Chen, J., Nagayama, T., Jin, K., Stetler, R. A., Zhu, R. L., Graham, S. H., and Simon, R. P. (1998) Induction of caspase-3-like protease may mediate delayed neuronal death in the hippocampus after transient cerebral ischemia. *J. Neurosci.,* **18,** 4914–4928.

Comi, G. (1997) Evoked potentials in diabetes mellitus. *Clin. Neurosci.,* **4,** 374–379.

Cracco, J., Castells, S., and Mark, E. (1984) Spinal somatosensory evoked potentials in juvenile diabetes. *Ann. Neurol.,* **15,** 55–58.

de Courten-Myers, G. M., Xi, G., Hwang, J. H., Dunn, R. S., Mills, A. S., Holland, S. K., Wagner, K. R., and Myers, R. E. (2000) Hypoglycemic brain injury: Potentiation from respiratory depression and injury aggravation from hyperglycemic treatment overshoots. *J. Cereb. Blood Flow Metab.,* **20,** 82–92.

Deary, I. J., Crawford, J. R., Hepburn, D. A., Langan, S. J., Blackmore, L. M., and Frier, B. M. (1993) Severe hypoglycemia and intelligence in adult patients with insulin-treated diabetes. *Diabetes,* **42,** 341–344.

DeJong, R. N. (1977) CNS manifestations of diabetes mellitus. *Postgrad. Med.,* **61,** 101–107.

Dey, J., Misra, A., Desai, N. G., Mahapatra, A. K., and Padma, M. V. (1997) Cognitive function in younger type II diabetes. *Diabetes Care,* **20,** 32–35.

Di Mario, U., Morano, S., Valle, E., and Pozzessere, G. (1995) Electrophysiological alterations of the central nervous system in diabetes mellitus. *Diabetes Metab. Rev.,* **11,** 259–277.

Donald, M. W., Erdahl, D. L., Surridge, D. H., Monga, T. N., Lawson, J. S., Bird, C. E., and Letemendia, F. J. (1984) Functional correlates of reduced central conduction velocity in diabetic subjects. *Diabetes,* **33,** 627–633.

Du, C., Hu, R., Csernansky, C. A., Hsu, C. Y., and Choi, D. W. (1996) Very delayed infarction after mild focal cerebral ischemia: A role for apoptosis? *J. Cereb. Blood Flow Metab.,* **16,** 195–201.

Endres, M., Namura, S., Shimizu-Sasamata, M., Waeber, C., Zhang, L., Gomez-Isla, T., Hyman, B. T., and Moskowitz, M. A. (1998) Attenuation of delayed neuronal death after mild focal ischemia in mice by inhibition of the caspase family. *J. Cereb. Blood Flow Metab.,* **18,** 238–247.

Fadeel, B., Orrenius, S., and Zhivotovsky, B. (1999) Apoptosis in human disease: A new skin for the old ceremony? *Biochem. Biophys. Res. Commun.,* **266,** 699–717.

Fernqvist-Forbes, E., Johansson, B. L., and Eriksson, M. J. (2001) Effects of C-peptide on forearm blood flow and brachial artery dilatation in patients with type 1 diabetes mellitus. *Acta Physiol. Scand.,* **172,** 159–165.

Flood, J. F., Mooradian, A. D., and Morley, J. E. (1990) Characteristics of learning and memory in streptozocin-induced diabetic mice. *Diabetes,* **39,** 1391–1398.

Forst, T., Kunt, T., Pfutzner, A., Beyer, J., and Wahren, J. (1998) New aspects on biological activity of C-peptide in IDDM patients. *Exp. Clin. Endocrinol. Diabetes.,* **106,** 270–276.

Gardoni, F., Kamal, A., Bellone, C., Biessels, G. J., Ramakers, G. M., Cattabeni, F., Gispent, W. H., and Di Luca, M. (2002) Effects of streptozotocin-diabetes on the hippocampal NMDA receptor complex in rats. *J. Neurochem.,* **80,** 438–447.

Garris, D. R. (1984) Diabetes-associated, ventromedial hypothalamic neuronal degeneration in the Chinese hamster. *Neurosci. Lett.,* **44,** 287–291.

Garris, D. R., Diani, A. R., Smith, C., and Gerritsen, G. C. (1982) Depopulation of the ventromedial hypothalamic nucleus in the diabetic Chinese hamster. *Acta Neuropathol (Berl.),* **56,** 63–66.

Gerozissis, K., Rouch, C., Lemierre, S., Nicolaidis, S., and Orosco, M. (2001) A potential role of central insulin in learning and memory related to feeding. *Cell. Mol. Neurobiol.,* **21,** 389–401.

Gispen, W. H., and Biessels, G. J. (2000) Cognition and synaptic plasticity in diabetes mellitus. *Trends Neurosci.,* **23,** 542–549.

Gisselsson, L., Smith, M. L., and Siesjö, B. K. (1999) Hyperglycemia and focal brain ischemia. *J. Cereb. Blood Flow Metab.,* **19,** 288–297.

Gold, A. E., Deary, I. J., Jones, R. W., O'Hare, J. P., Reckless, J. P., and Frier, B. M. (1994) Severe deterioration in cognitive function and personality in five patients with long-standing diabetes: A complication of diabetes or a consequence of treatment? *Diabet. Med.,* **11,** 499–505.

Gorman, A. M., McGowan, A., O'Neill, C., and Cotter, T. (1996) Oxidative stress and apoptosis in neurodegeneration. *J. Neurol. Sci.,* **139 (Suppl),** 45–52.

Gradman, T. J., Laws, A., Thompson, L. W., and Reaven, G. M. (1993) Verbal learning and/or memory improves with glycemic control in older subjects with non-insulin-dependent diabetes mellitus. *J. Am. Geriatr. Soc.,* **41,** 1305–1312.

Harkins, S. W., Gardner, D. F., and Anderson, R. A. (1985) Auditory and somatosensory far-field evoked potentials in diabetes mellitus. *Int. J. Neurosci.,* **28,** 41–47.

Hassing, L. B., Johansson, B., Nilsson, S. E., Berg, S., Pedersen, N. L., Gatz, M., and McClearn, G. (2002) Diabetes mellitus is a risk factor for vascular dementia, but not for Alzheimer's disease: A population-based study of the oldest old. *Int. Psychogeriatr.,* **14,** 239–248.

Holmes, C. S., and Richman, L. C. (1985) Cognitive profiles of children with insulin-dependent diabetes. *J. Dev. Behav. Pediatr.,* **6,** 323–326.

Huang, D. Y., Richter, K., Breidenbach, A., and Vallon, V. (2002) Human C-peptide acutely lowers glomerular hyperfiltration and proteinuria in diabetic rats: A dose-response study. *Naunyn Schmiedebergs Arch Pharmacol.,* **365,** 67–73.

Ido, Y., Vindigni, A., Chang, K., Stramm, L., Chance, R., Heath, W. F., DiMarchi, R. D., Di Cera, E., and Williamson, J. R. (1997) Prevention of vascular and neural dysfunction in diabetic rats by C-peptide. *Science,* **277,** 563–566.

Jakobsen, J., Sidenius, P., Gundersen, H. J., and Østerby, R. (1987) Quantitative changes of cerebral neocortical structure in insulin-treated long-term streptozocin-induced diabetes in rats. *Diabetes,* **36,** 597–601.

Johansson, B. L., Borg, K., Fernqvist-Forbes, E., Odergren, T., Remahl, S., and Wahren, J. (1996) C-peptide improves autonomic nerve function in IDDM patients. *Diabetologia,* **39,** 687–695.

Johansson, B. L., Sjöberg, S., and Wahren, J. (1992) The influence of human C-peptide on renal function and glucose utilization in type 1 (insulin-dependent) diabetic patients. *Diabetologia,* **35,** 121–128.

Kamal, A., Biessels, G. J., Duis, S. E., and Gispen, W. H. (2000) Learning and hippocampal synaptic plasticity in streptozotocin-diabetic rats: Interaction of diabetes and ageing. *Diabetologia,* **43,** 500–506.

Kamal, A., Biessels, G. J., Urban, I. J., and Gispen, W. H. (1999) Hippocampal synaptic plasticity in streptozotocin-diabetic rats:

Impairment of long-term potentiation and facilitation of long-term depression. *Neuroscience,* **90,** 737–745.

Kaminski, K. A., Bonda, T. A., Korecki, J., and Musial, W. J. (2002) Oxidative stress and neutrophil activation—the two keystones of ischemia/reperfusion injury. *Int. J. Cardiol.,* **86,** 41–59.

Kothari, V., Stevens, R. J., Adler, A. I., Stratton, I. M., Manley, S. E., Neil, H. A., and Holman, R. R. (2002) UKPDS 60: Risk of stroke in type 2 diabetes estimated by the UK Prospective Diabetes Study risk engine. *Stroke,* **33,** 1776–1781.

Kovacs, M., Goldston, D., Obrosky, D. S., and Bonar, L. K. (1997) Psychiatric disorders in youths with IDDM: Rates and risk factors. *Diabetes Care,* **20,** 36–44.

Kramer, L., Fasching, P., Madl, C., Schneider, B., Damjancic, P., Waldhäusl, W., Irsigler, K., and Grimm, G. (1998) Previous episodes of hypoglycemic coma are not associated with permanent cognitive brain dysfunction in IDDM patients on intensive insulin treatment. *Diabetes,* **47,** 1909–1914.

Kulikov, A. V., Arkhipova, L. V., Tretyak, T. M., and Bragin, A. G. (1986) Serotonin and norepinephrine content in brain structures of rats with experimental and transplantation-compensated diabetes. *J. Hirnforsch.,* **27,** 495–499.

Kurita, A., Mochio, S., and Isogai, Y. (1995) Changes in auditory P300 event-related potentials and brainstem evoked potentials in diabetes mellitus. *Acta Neurol. Scand.,* **92,** 319–323.

Laing, S. P., Swerdlow, A. J., Carpenter, L. M., Slater, S. D., Burden, A. C., Botha, J. L., Morris, A. D., Waugh, N. R., Gatling, W., Gale, E. A., Patterson, C. C., Qiao, Z., and Keen, H. (2003) Mortality from cerebrovascular disease in a cohort of 23000 patients with insulin-treated diabetes. *Stroke,* **34,** 418–421.

Lee-Kwon, W., Park, D., Baskar, P. V., Kole, S., and Bernier, M. (1998) Antiapoptotic signaling by the insulin receptor in Chinese hamster ovary cells. *Biochemistry,* **37,** 15747–15757.

Lehto, S., Ronnemaa, T., Pyorala, K., and Laakso, M. (1996) Predictors of stroke in middle-aged patients with non-insulin-dependent diabetes. *Stroke,* **27,** 63–68.

LeMay, D. R., Gehua, L., Zelenock, G. B., and D'Alecy, L. G. (1988) Insulin administration protects neurologic function in cerebral ischemia in rats. *Stroke,* **19,** 1411–1419.

Li, L., Oshida, Y., Kusunoki, M., Yamanouchi, K., Johansson, B. L., Wahren, J., and Sato, Y. (1999) Rat C peptide I and II stimulate glucose utilization in STZ-induced diabetic rats. *Diabetologia,* **42,** 958–964.

Li, P. A., Gisselsson, L., Keuker, J., Vogel, J., Smith, M. L., Kuschinsky, W., and Siesjö, B. K. (1998) Hyperglycemia-exaggerated ischemic brain damage following 30 min of middle cerebral artery occlusion is not due to capillary obstruction. *Brain Res.,* **804,** 36–44.

Li, X., Aou, S., Hori, T., and Oomura, Y. (2002a) Spatial memory deficit and emotional abnormality in OLETF rats. *Physiol. Behav.,* **75,** 15–23.

Li, Y., Sharov, V. G., Jiang, N., Zaloga, C., Sabbah, H. N., and Chopp, M. (1995) Ultrastructural and light microscopic evidence of apoptosis after middle cerebral artery occlusion in the rat. *Am. J. Pathol.,* **146,** 1045–1051.

Li, Z. G., Qiang, X., Sima, A. A. F., and Grunberger, G. (2001) C-peptide attenuates protein tyrosine phosphatase activity and enhances glycogen synthesis in L6 myoblasts. *Biochem. Biophys. Res. Commun.,* **280,** 615–619.

Li, Z. G., Zhang, W., Grunberger, G., and Sima, A. A. F. (2002b) Hippocampal neuronal apoptosis in type 1 diabetes. *Brain Res.,* **946,** 221–231.

Li, Z. G., Zhang, W., and Sima, A. A. F. (2002c) C-peptide prevents hippocampal apoptosis in type 1 diabetes. *Int. J. Exp. Diabetes Res.,* **3,** 241–245.

Li, Z. G., Zhang, W., and Sima, A. A. F. (2003) C-peptide enhances insulin-mediated cell growth and protection against high glucose-induced apoptosis in SH-SY5Y cells. *Diabetes Metab. Res. Rev.,* **19,** 375–385.

Lipinski, B. (2001) Pathophysiology of oxidative stress in diabetes mellitus. *J. Diabetes Complications,* **15,** 203–210.

Love, S. (1999) Oxidative stress in brain ischemia. *Brain Pathol.,* **9,** 119–131.

Lowe, L. P., Tranel, D., Wallace, R. B., and Welty, T. K. (1994) Type II diabetes and cognitive function. A population-based study of Native Americans. *Diabetes Care,* **17,** 891–896.

Luesse, H. G., Schiefer, J., Spruenken, A., Puls, C., Block, F., and Kosinski, C. M. (2001) Evaluation of R6/2 HD transgenic mice for therapeutic studies in Huntington's disease: Behavioral testing and impact of diabetes mellitus. *Behav. Brain Res.,* **126,** 185–195.

Lunetta, M., Damanti, A. R., Fabbri, G., Lombardo, M., Di Mauro, M., and Mughini, L. (1994) Evidence by magnetic resonance imaging of cerebral alterations of atrophy type in young insulin-dependent diabetic patients. *J. Endocrinol. Invest.,* **17,** 241–245.

Luo, Y., Cao, G., Pei, W., O'Horo, C., Graham, S. H., and Chen, J. (2002) Induction of caspase-activated deoxyribonuclease activity after focal cerebral ischemia and reperfusion. *J. Cereb. Blood Flow Metab.,* **22,** 15–20.

Luse, S. A. (1970) The ultrastructure of the brain in the diabetic Chinese hamster with special reference to synaptic abnormalities. *Electroencephalogr. Clin. Neurophysiol.,* **29,** 410.

Lustman, P. J., Griffith, L. S., and Clouse, R. E. (1988) Depression in adults with diabetes. Results of 5-yr follow-up study. *Diabetes Care,* **11,** 605–612.

MacKnight, C., Rockwood, K., Awalt, E., and McDowell, I. (2002) Diabetes mellitus and the risk of dementia, Alzheimer's disease and vascular cognitive impairment in the Canadian Study of Health and Aging. *Dement. Geriatr. Cogn. Disord.,* **14,** 77–83.

Madl, C., Grimm, G., Kramer, L., Koppensteiner, R., Hirschl, M., Yeganehfar, W., Hirschl, M. M., Ugurluoglu, A., Schneider, B., and Ehringer, H. (1994) Cognitive brain function in non-demented patients with low-grade and high-grade carotid artery stenosis. *Eur. J. Clin. Invest.,* **24,** 559–564.

Manson, J. E., Colditz, G. A., Stampfer, M. J., Willett, W. C., Krolewski, A. S., Rosner, B., Arky, R. A., Speizer, F. E., and Hennekens, C. H. (1991) A prospective study of maturity-onset diabetes mellitus and risk of coronary heart disease and stroke in women. *Arch. Intern. Med.,* **151,** 1141–1147.

McCarthy, A. M., Lindgren, S., Mengeling, M. A., Tsalikian, E., and Engvall, J. C. (2002) Effects of diabetes on learning in children. *Pediatrics,* **109,** E9.

Mercuri, F., Quagliaro, L., and Ceriello, A. (2000) Oxidative stress evaluation in diabetes. *Diabetes Technol. Ther.,* **2,** 589–600.

Miles, W. R., and Root, H. F. (1922) Psychologic tests applied to diabetic patients. *Arch. Int. Med.,* **30,** 767–777.

Mochizuki, Y., Oishi, M., Hayakawa, Y., Matsuzaki, M., and Takasu, T. (1998) Improvement of P300 latency by treatment in non-insulin-dependent diabetes mellitus. *Clin. Electroencephalogr.,* **29,** 194–196.

Mooradian, A. D. (1997) Central nervous system complications of diabetes mellitus—a perspective from the blood-brain barrier. *Brain Res. Brain Res. Rev.,* **23,** 210–218.

Mooradian, A. D., Perryman, K., Fitten, J., Kavonian, G. D., and Morley, J. E. (1988) Cortical function in elderly non-insulin dependent diabetic patients. Behavioral and electrophysiologic studies. *Arch. Intern. Med.,* **148,** 2369–2372.

Mukai, N., Hori, S., and Pomeroy, M. (1980) Cerebral lesions in rats with streptozotocin-induced diabetes. *Acta Neuropathol. (Berl.),* **51,** 79–84.

Muranyi, M., Fujioka, M., He, Q., Han, A., Yong, G., Csiszar, K., and Li, P. A. (2003) Diabetes activates cell death pathway after transient focal cerebral ischemia. *Diabetes,* **52,** 481–486.

Namura, S., Zhu, J., Fink, K., Endres, M., Srinivasan, A., Tomaselli, K. J., Yuan, J., and Moskowitz, M. A. (1998) Activation and cleavage of caspase-3 in apoptosis induced by experimental cerebral ischemia. *J. Neurosci.,* **18,** 3659–3668.

Nicotera, P., Ankarcrona, M., Bonfoco, E., Orrenius, S., and Lipton, S. A. (1997) Neuronal necrosis and apoptosis: Two distinct events induced by exposure to glutamate or oxidative stress. *Adv. Neurol.,* **72,** 95–101.

Opara, E. C. (2002) Oxidative stress, micronutrients, diabetes mellitus and its complications. *J. R. Soc. Health.,* **122,** 28–34.

Orrenius, S. (1995) Apoptosis: Molecular mechanisms and implications for human disease. *J. Intern. Med.,* **237,** 529–536.

Ott, A., Stolk, R. P., van Harskamp, F., Pols, H. A., Hofman, A., and Breteler, M. M. (1999) Diabetes mellitus and the risk of dementia: The Rotterdam Study. *Neurology,* **53,** 1937–1942.

Parisi, V., and Uccioli, L. (2001) Visual electrophysiological responses in persons with type 1 diabetes. *Diabetes Metab. Res. Rev.,* **17,** 12–18.

Perlmuter, L. C., Hakami, M. K., Hodgson-Harrington, C., Ginsberg, J., Katz, J., Singer, D. E., and Nathan, D. M. (1984) Decreased cognitive function in aging non-insulin-dependent diabetic patients. *Am. J. Med.,* **77,** 1043–1048.

Perros, P., Deary, I. J., Sellar, R. J., Best, J. J., and Frier, B. M. (1997) Brain abnormalities demonstrated by magnetic resonance imaging in adult IDDM patients with and without a history of recurrent severe hypoglycemia. *Diabetes Care,* **20,** 1013–1018.

Picton, T. W. (1992) The P300 wave of the human event-related potential. *J. Clin. Neurophysiol,* **9,** 456–479.

Pierson, C. R., Zhang, W., and Sima, A. A. F. (2003) Proinsulin C-peptide replacement in type 1 diabetic BB/Wor-rats prevents deficits in nerve fiber regeneration. *J. Neuropathol. Exp. Neurol.,* **62,** 765–779.

Pietravalle, P., Morano, S., Cristina, G., Mancuso, M., Valle, E., Annulli, M. A., Tomaselli, M., Pozzessere, G., and Di Mario, U. (1993) Early complications in type 1 diabetes: Central nervous system alterations preceded kidney abnormalities. *Diabetes Res. Clin. Pract.,* **21,** 143–154.

Popkin, M. K., Callies, A. L., Lentz, R. D., Colon, E. A., and Sutherland, D. E. (1988) Prevalence of major depression, simple phobia, and other psychiatric disorders in patients with long-standing type I diabetes mellitus. *Arch. Gen. Psychiatry,* **45,** 64–68.

Pozzessere, G., Valle, E., de Crignis, S., Cordischi, V. M., Fattapposta, F., Rizzo, P. A., Pietravalle, P., Cristina, G., Morano, S., and di Mario, U. (1991) Abnormalities of cognitive functions in IDDM revealed by P300 event-related potential analysis. Comparison with short-latency evoked potentials and psychometric tests. *Diabetes,* **40,** 952–958.

Reaven, G. M., Thompson, L. W., Nahum, D., and Haskins, E. (1990) Relationship between hyperglycemia and cognitive function in older NIDDM patients. *Diabetes Care,* **13,** 16–21.

Reske-Nielsen, E., and Lundbaek, K. (1964) Diabetic encephalopathy. Diffuse and focal lesions of the brain in long-term diabetics. *Acta Neurol. Scand.,* **Suppl. 4,** 273–290.

Reske-Nielsen, E., Lundbaek, K., and Rafeisen, Q. J. (1965) Pathological changes in the central and peripheral nervous system of young long-term diabetics I. Diabetic encephalopathy. *Diabetologia,* **1,** 233–241.

Rolfe, M., Ephraim, G. G., Lincoln, D. C., and Huddle, K. R. (1995) Hyperosmolar non-ketotic diabetic coma as a cause of emergency hyperglycaemic admission to Baragwanath Hospital. *S. Afr. Med. J.,* **85,** 173–176.

Rubini, R., Biasiolo, F., Fogarolo, F., Magnavita, V., Martini, A., and Fiori, M. G. (1992) Brainstem auditory evoked potentials in rats with streptozotocin-induced diabetes. *Diabetes Res. Clin. Pract.,* **16,** 19–25.

Ryan, C. M. (1988) Neurobehavioral complications of type I diabetes. Examination of possible risk factors. *Diabetes Care,* **11,** 86–93.

Ryan, C. M., and Geckle, M. (2000a) Why is learning and memory dysfunction in Type 2 diabetes limited to older adults? *Diabetes Metab. Res. Rev.,* **16,** 308–315.

Ryan, C. M., and Geckle, M. O. (2000b) Circumscribed cognitive dysfunction in middle-aged adults with type 2 diabetes. *Diabetes Care,* **23,** 1486–1493.

Ryan, C. M., and Williams, T. M. (1993) Effects of insulin-dependent diabetes on learning and memory efficiency in adults. *J. Clin. Exp. Neuropsychol.,* **15,** 685–700.

Ryan, C. M., Williams, T. M., Finegold, D. N., and Orchard, T. J. (1993) Cognitive dysfunction in adults with type 1 (insulin-dependent) diabetes mellitus of long duration: Effects of recurrent hypoglycaemia and other chronic complications. *Diabetologia,* **36,** 329–334.

Samnegard, B., Jacobson, S. H., Jaremko, G., Johansson, B. L., and Sjöquist, M. (2001) Effects of C-peptide on glomerular and renal size and renal function in diabetic rats. *Kidney Int.,* **60,** 1258–1265.

Sastre, J., Pallardo, F. V., and Vina, J. (2000) Mitochondrial oxidative stress plays a key role in aging and apoptosis. *IUBMB Life,* **49,** 427–435.

Schoenle, E. J., Schoenle, D., Molinari, L., and Largo, R. H. (2002) Impaired intellectual development in children with type I diabetes: Association with HbA(1c), age at diagnosis and sex. *Diabetologia,* **45,** 108–114.

Schulz, J. B., Weller, M., and Moskowitz, M. A. (1999) Caspases as treatment targets in stroke and neurodegenerative diseases. *Ann. Neurol.,* **45,** 421–429.

Schwartz, M. W., Figlewicz, D. P., Baskin, D. G., Woods, S. C., and Porte, D., Jr. (1992) Insulin in the brain: A hormonal regulator of energy balance. *Endocr. Rev.,* **13,** 387–414.

Sima, A. A. F. (2003a) New insights into the metabolic and molecular basis for diabetic neuropathy. *Cell. Mol. Life Sci.,* **60,** 2445–2464.

Sima, A. A. F. (2003b) C-peptide and diabetic neuropathy. *Expert. Opin. Invest. Drugs,* **12,** 1471–1488.

Sima, A. A. F., Zhang, W. X., Cherian, P. V., and Chakrabarti, S. (1992) Impaired visual evoked potential and primary axonopathy of the optic nerve in the diabetic BB/W-rat. *Diabetologia,* **35,** 602–607.

Sima, A. A. F., Zhang, W., Sugimoto, K., Henry, D., Li, Z.-G., Wahren, J., and Grunberger, G. (2001) C-peptide prevents and improves chronic type 1 diabetic neuropathy in the BB/Wor-rat. *Diabetologia,* **44,** 889–897.

Sinclair, A. J., Girling, A. J., and Bayer, A. J. (2000) Cognitive dysfunction in older subjects with diabetes mellitus: Impact on diabetes self-management and use of care services. All Wales Research into Elderly (AWARE) Study. *Diabetes Res. Clin. Pract.,* **50,** 203–212.

Stamler, J., Vaccaro, O., Neaton, J. D., and Wentworth, D. (1993) Diabetes, other risk factors, and 12-yr cardiovascular mortality for men screened in the Multiple Risk Factor Intervention Trial. *Diabetes Care,* **16,** 434–444.

Starr, J. M., Wardlaw, J., Ferguson, K., MacLullich, A., Deary, I. J., and Marshall, I. (2003) Increased blood-brain barrier permeability in type II diabetes demonstrated by gadolinium magnetic resonance imaging. *J. Neurol. Neurosurg. Psychiatry,* **74,** 70–76.

Steiner, D. F., and Rubenstein, A. H. (1997) Proinsulin C-peptide—biological activity? *Science,* **277,** 531–532.

Strachan, M. W., Deary, I. J., Ewing, F. M., and Frier, B. M. (1997) Is type II diabetes associated with an increased risk of cognitive dysfunction? A critical review of published studies. *Diabetes Care,* **20,** 438–445.

Sullivan, J. M., Alousi, S. S., Hikade, K. R., Bahu, N. J., Rafols, J. A., Krause, G. S., and White, B. C. (1999) Insulin induces dephosphorylation of eukaryotic initiation factor 2alpha and restores protein synthesis in vulnerable hippocampal neurons after transient brain ischemia. *J. Cereb. Blood Flow Metab.,* **19,** 1010–1019.

Terry, B., Cannon, J., Guong, L., Wands, J., and SM., d.l.M. (2001) Abnormalities in insulin, IGF-I, and corresponding receptor (R) expression in Alzheimer disease. *J. Neuropathol. Exp. Neurol.,* **60,** 546.

Thompson, C. B. (1995) Apoptosis in the pathogenesis and treatment of disease. *Science,* **267,** 1456–1462.

Ting, J. Y. (2001) Hyperosmolar diabetic non-ketotic coma, hyperkalaemia and an unusual near death experience. *Eur. J. Emerg. Med.,* **8,** 57–63.

Trulson, M. E., Jacoby, J. H., and MacKenzie, R. G. (1986) Streptozotocin-induced diabetes reduces brain serotonin synthesis in rats. *J. Neurochem.,* **46,** 1068–1072.

Tun, P. A., Nathan, D. M., and Perlmuter, L. C. (1990) Cognitive and affective disorders in elderly diabetics. *Clin. Geriatr. Med.,* **6,** 731–746.

Uberall, M. A., Renner, C., Edl, S., Parzinger, E., and Wenzel, D. (1996) VEP and ERP abnormalities in children and adolescents with prepubertal onset of insulin-dependent diabetes mellitus. *Neuropediatrics,* **27,** 88–93.

Vannucci, R. C., and Vannucci, S. J. (2001) Hypoglycemic brain injury. *Semin. Neonatol.,* **6,** 147–155.

Varsik, P., Kucera, P., Buranova, D., and Balaz, M. (2001) Is the spinal cord lesion rare in diabetes mellitus? Somatosensory evoked potentials and central conduction time in diabetes mellitus. *Med. Sci. Monit.,* **7,** 712–715.

Verrotti, A., Lobefalo, L., Trotta, D., Della Loggia, G., Chiarelli, F., Luigi, C., Morgese, G., and Gallenga, P. (2000) Visual evoked potentials in young persons with newly diagnosed diabetes: A long-term follow-up. *Dev. Med. Child. Neurol.,* **42,** 240–244.

Voll, C. L., and Auer, R. N. (1991a) Insulin attenuates ischemic brain damage independent of its hypoglycemic effect. *J. Cereb. Blood Flow Metab.,* **11,** 1006–1014.

Voll, C. L., and Auer, R. N. (1991b) Postischemic seizures and necrotizing ischemic brain damage: Neuroprotective effect of postischemic diazepam and insulin. *Neurology,* **41,** 423–428.

Wahren, J., and Johansson, B. L. (1998) Ernst-Friedrich-Pfeiffer Memorial Lecture. New aspects of C-peptide physiology. *Horm. Metab. Res.,* **30,** A2–A5.

Wahren, J., Johansson, B. L., and Wallberg-Henriksson, H. (1994) Does C-peptide have a physiological role? *Diabetologia,* **37 (Suppl 2),** S99–S107.

Wahren, J., Johansson, B. L., Wallberg-Henriksson, H., Linde, B., Fernqvist-Forbes, E., and Zierath, J. R. (1996) C-peptide revisited—new physiological effects and therapeutic implications. *J. Intern. Med.,* **240,** 115–124.

Worrall, G., Moulton, N., and Briffett, E. (1993) Effect of type II diabetes mellitus on cognitive function. *J. Fam. Pract.,* **36,** 639–643.

Wu, W., Oshida, Y., Yang, W. P., Li, L., Ohsawa, I., Sato, J., Iwao, S., Johansson, B. L., Wahren, J., and Sato, Y. (1996) Effect of C-peptide administration on whole body glucose utilization in STZ-induced diabetic rats. *Acta Physiol. Scand.,* **157,** 253–258.

Wuarin, L., Namdev, R., Burns, J. G., Fei, Z. J., and Ishii, D. N. (1996) Brain insulin-like growth factor-II mRNA content is reduced in insulin-dependent and non-insulin-dependent diabetes mellitus. *J. Neurochem.,* **67,** 742-751.

Yager, J. Y. (2002) Hypoglycemic injury to the immature brain. *Clin. Perinatol.,* **29,** 651–674.

Zhang, W., Yorek, M., Pierson, C. R., Murakawa, Y., Breidenbach, A., and Sima, A. A. F. (2001) Human C-peptide dose dependently prevents early neuropathy in the BB/Wor-rat. *Int. J. Exp. Diabetes Res.,* **2,** 187–193.

Zhao, W. Q., and Alkon, D. L. (2001) Role of insulin and insulin receptor in learning and memory. *Mol. Cell. Endocrinol.,* **177,** 125–134.

Ziegler, O., Guerci, B., Algan, M., Lonchamp, P., Weber, M., and Drouin, P. (1994) Improved visual evoked potential latencies in poorly controlled diabetic patients after short-term strict metabolic control. *Diabetes Care,* **17,** 1141–1147.

Zierath, J. R., Galuska, D., Johansson, B. L., and Wallberg-Henriksson, H. (1991) Effect of human C-peptide on glucose transport in in vitro incubated human skeletal muscle. *Diabetologia,* **34,** 899–901.

Zierath, J. R., Handberg, A., Tally, M., and Wallberg-Henriksson, H. (1996) C-peptide stimulates glucose transport in isolated human skeletal muscle independent of insulin receptor and tyrosine kinase activation. *Diabetologia,* **39,** 306–313.

